# P-1869. When Expertise Matters: ID Consultation is Associated with Lower Mortality in Serratia marcescens Bacteremia

**DOI:** 10.1093/ofid/ofaf695.2038

**Published:** 2026-01-11

**Authors:** Donna R Burgess, Katie B Olney, David S Burgess

**Affiliations:** UK HealthCare, Lexington, KY; University of Kentucky HealthCare, Lexington, KY; University of Kentucky, Lexington, KY

## Abstract

**Background:**

*Serratia marcescens* is an opportunistic gram-negative pathogen increasingly associated with healthcare-associated infections. Data on outcomes and factors influencing mortality in *S. marcescens* bacteremia are limited.

**Methods:**

We conducted a retrospective cohort study of all inpatients with an initial positive blood culture for *S. marcescens* at UK HealthCare from July 1, 2022, to December 31, 2024. Among 970 patients with bacteremia, 61 (6.3%) had *S. marcescens*. Clinical characteristics, management strategies, and outcomes were analyzed. Data were obtained through manual chart review and the Center for Clinical and Translational Science (CCTS) database. Predictors of in-hospital mortality were evaluated using univariate analysis.

**Results:**

Among 61 patients with *S. marcescens* bacteremia, the median (IQR) age was 58 years (44, 68), and 58.1% were male. Over half of infections (53.2%) were hospital-acquired. Common comorbidities included COPD (45.2%), diabetes with complications (35.5%), congestive heart failure (CHF) (43.5%), and peripheral vascular disease (41.9%). ICU admission occurred in 43.5% of patients. The median hospital length of stay was 17 days (10, 35), with ICU length of stay of 8.4 days (3.8, 15.3). In-hospital mortality was 27.4%, and 90-day readmission occurred in 38.7% of patients.

An Infectious Diseases (ID) consult was obtained in 54.8% of cases. ID involvement was significantly more frequent in non-ICU patients (77.1%, 27/35) compared to ICU patients (25.9%, 7/27; p < 0.001). Mortality was substantially lower in patients who received an ID consult: 14.7% (5/34) vs 57.1% (16/28) without consult (p = 0.013). Other significant predictors of mortality included ICU admission (p = 0.039), qPitt score (p < 0.001), Charlson Comorbidity Index (p = 0.042), and SOFA score (p < 0.001).

**Conclusion:**

*Serratia marcescens* accounted for a small proportion of bacteremia cases but was associated with high mortality. ID consultation was associated with a four-fold reduction in mortality yet was markedly underutilized in critically ill patients. These findings highlight a significant opportunity to improve outcomes through earlier and more consistent ID involvement, especially in the ICU setting.

**Disclosures:**

All Authors: No reported disclosures

